# Effect of *Adiantum philippense* Extract on Biofilm Formation, Adhesion With Its Antibacterial Activities Against Foodborne Pathogens, and Characterization of Bioactive Metabolites: An *in vitro-in silico* Approach

**DOI:** 10.3389/fmicb.2020.00823

**Published:** 2020-05-13

**Authors:** Mohd Adnan, Mitesh Patel, Sumukh Deshpande, Mousa Alreshidi, Arif Jamal Siddiqui, Mandadi Narsimha Reddy, Noumi Emira, Vincenzo De Feo

**Affiliations:** ^1^Department of Biology, College of Science, University of Ha’il, Ha’il, Saudi Arabia; ^2^Department of Biosciences, Bapalal Vaidya Botanical Research Centre, Veer Narmad South Gujarat University, Surat, India; ^3^Central Biotechnology Services, College of Biomedical and Life Sciences, Cardiff University, Cardiff, United Kingdom; ^4^Department of Pharmacy, University of Salerno, Via Giovanni Paolo II, Fisciano, Italy

**Keywords:** biofilms, antibacterial, foodborne pathogens, *Adiantum philippense*, molecular docking, phytochemical analysis

## Abstract

*Adiantum philippense* (*A. philippense*), an ethnomedicinally important fern, has become an interesting herb in the search for novel bioactive metabolites, which can also be used as therapeutic agents. Primarily, in this study, *A. philippense* crude extract was screened for its phytochemical constituents, antagonistic potential, and effect on bacterial adhesion and biofilm formation against common food pathogens. Phytochemical profiling of *A. philippense* was carried out by using High Resolution-Liquid Chromatography and Mass Spectroscopy (HR-LCMS) followed by antibacterial activity via agar cup/well diffusion, broth microdilution susceptibility methods, and growth curve analysis. Antibiofilm potency and efficacy were assessed on the development, formation, and texture of biofilms through light microscopy, fluorescent microscopy, scanning electron microscopy, and the assessment of exopolysaccharide production. Correspondingly, a checkerboard test was performed to evaluate the combinatorial effect of *A. philippense* and chloramphenicol. Lastly, molecular docking studies of identified phytochemicals with adhesin proteins of tested food pathogens, which helps the bacteria in surface attachment and leads to biofilm formation, were assessed. *A. philippense* crude extract was found to be active against all tested food pathogens, displaying the rapid time-dependent kinetics of bacterial killing. *A. philippense* crude extract also impedes the biofilm matrix by reducing the total content of exopolysaccharide, and, likewise, the microscopic images revealed a great extent of disruption in the architecture of biofilms. A synergy was observed between *A. philippense* crude extract and chloramphenicol for *E. coli, S. aureus*, and *P. aeruginosa*, whereas an additive effect was observed for *S. flexneri*. Various bioactive phytochemicals were categorized from *A. philippense* crude extract using HR-LCMS. The molecular docking of these identified phytochemicals was interrelated with the active site residues of adhesin proteins, IcsA, Sortase A, OprD, EspA, and FimH from *S. flexneri, S. aureus, P. aeruginosa*, and *E. coli*, respectively. Thus, our findings represent the bioactivity and potency of *A. philippense* crude extract against food pathogens not only in their planktonic forms but also against/in biofilms for the first time. We have also correlated these findings with the possible mechanism of biofilm inhibition via targeting adhesin proteins, which could be explored further to design new bioactive compounds against biofilm producing foodborne bacterial pathogens.

## Introduction

Since the conception of human civilization, foodborne diseases have been a concern for mankind. The foremost cause of food poisoning and foodborne diseases are foodborne pathogens that consequently pose a grave risk to food safety (Oliver et al., 2005). Over the last few years, the number of diseases caused by foodborne pathogens has been on an increase, and this has ultimately turned into a primary and serious global health issue (Zhao et al., 2017). Food contaminating pathogens have garnered significant attention, as they are currently the cause of remarkable mortality and morbidity numbers with a rate of 420,000 deaths per year (World Health Organization [WHO], 2018). As per the data from Centre for Disease Control and Prevention (CDC), *Escherichia coli*, *Staphylococcus aureus, Shigella flexneri, Listeria* spp., *Clostridium perfringens, Campylobacter* spp., and *Salmonella* spp. are a few of the pathogens that cause food poisoning (Scallan et al., 2011). The most ordinary symptoms of these food pathogens are diarrhea, vomiting, abdominal cramps, fatigue, nausea, and fever. These pathogens can contaminate foodstuffs at any stage during processing, distribution, and storage. Therefore, it is extremely crucial that we control the growth and development of food pathogens, though removal of these organisms is challenging since they are capable of forming biofilms on a variety of planes (Bazargani, 2016).

Biofilms are three-dimensional microbial communities that are surface-attached, compact, structured, and embedded in a self-produced extracellular polymeric substances matrix comprising of proteins, polysaccharides, and other molecules (Adnan et al., 2010; Adnan et al., 2011, 2017c; Di Ciccio et al., 2015). Foodborne pathogens are usually proficient in adhering to various type of surfaces (inert or living) and forming biofilms. Once the biofilm is formed, the bacteria inside are less susceptible to antibiotics and other chemical substances than their counterparts, planktonic cells (Adnan et al., 2010, 2017c). This increases the resistance of biofilm producing bacterial cells against antimicrobial agents and reduces the efficacy for biofilm-associated treatment (Meesilp and Mesil, 2019). The foodborne bacteria in their planktonic forms cause serious health issues and safety concerns, and, when they assemble in the form of biofilms, the problem is much more threatening (Ribeiro et al., 2016). Not only are the bacterial cells in biofilm resistant to antibiotics, they are also able to defend themselves against a number of physico-chemical aggressions, including acidity, salinity, heavy metals, ultraviolet light, and phagocytosis (Lebeaux et al., 2014). Foodborne bacterial biofilms are an extensive threat to dairy and other food industries as an origin of contamination; they can lead to severe hygienic complications and great economic loss. Furthermore, the origins of a lot of food poisoning epidemics have been linked to biofilms forming pathogens in the meat, poultry, dairy, and ready-to-eat food industries (Srey et al., 2014). The reason for this is that biofilms are tough to exterminate once they are formed (Wu et al., 2015).

In view of the fact, biofilm formation poses a great risk worldwide for marine and oceanic industries, food and dairy industries, and, most importantly, public health (Adnan et al., 2018). Treating biofilms is a global challenge that necessitates the invention of novel natural bioactive molecules against foodborne pathogenic bacteria. The necessity for natural bioactive compounds – as opposed to the chemically synthesized ones – is due to dealings with food industries. As a result, we have evaluated the antibacterial and antibiofilm potential of *A. philippense* (Adiantaceae) crude extract. *A. philippense* is a medicinally treasured fern with several curative properties. These days, plant-derived extracts are extensively considered due to their lack of side effects, and many are currently being used traditionally as ethnomedicine for the prevention and treatment of different types of infections (Adnan et al., 2017c). In India, *A. philippense* is commonly utilized by the locals and tribal groups for the treatment of several medical conditions, such as epileptic fits, fever, ulcers, blood diseases, erysipelas, dysentery, rabies, febrile affection, emaciation or cachexia, muscular pain atrophy, paralysis, pimple, wounds, and elephantiasis (Chopra et al., 1956; Asolkar et al., 1992; Rashid, 1999). The presence of phenols, terpenoids, flavonoids, and carbohydrates has been observed, and this resulted from the phytochemical analysis of this plant. Thus, availability of these compounds gives this fern potency to act as a healer (Ambasta, 1986; Sant et al., 2013), but there is a lack of research into its detailed phytochemistry and constituent analysis.

Previous studies on medicinal plants have described the antibiofilm potential of phytochemicals via repression of quorum sensing (Davies and Webb, 1998). Many known and popular quorum-sensing inhibitors are different types of different phytochemicals, such as flavonols, flavonoids, phenols, and flavonones (Keen et al., 2001; Vandeputte et al., 2011; Truchado et al., 2012; Sarabhai et al., 2013). Similarly, such kinds of phytochemicals are also known for inhibition of bacterial adhesion and for repression of genes associated with the formation of biofilm. This study was therefore aimed at evaluating the effect of *A. philippense* phytochemicals on biofilm formation adhesion with its antibacterial properties against common food pathogens *Escherichia coli (E. coli), Staphylococcus aureus (S. aureus), Shigella flexneri (S. flexneri)*, and *Pseudomonas aeruginosa (P. aeruginosa). In vitro* and *in silico* approaches were considered to achieve the targeted aims because computational tools have extended their reach into different realms of scientific research. Using a simulated molecular docking technique to provide comprehensive insight into the molecular mechanisms of biological processes is an additional approach that has been followed in this study to analyze the anti-adhesion ability of *A. philippense*. Moreover, the influence of molecular docking is also vital in validating novel lead compounds and will further reveal the predicted mode of binding between identified derivatives with their receptor site.

## Materials and Methods

### Plant Material

**FIGURE 1:**
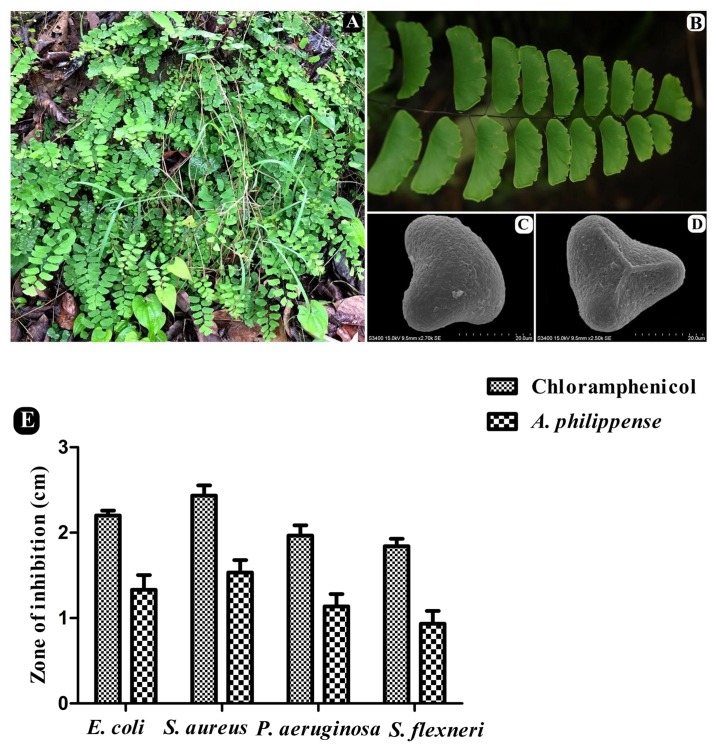
*A. philippense* plant and its antibacterial activity. **(A)** Plants in wild; **(B)** close up of frond; **(C)** distal face of spore under SEM; **(D)** proximal face of spore under SEM; and **(E)** antibacterial activity against *E. coli*, *S. aureus*, *P. aeruginosa*, and *S. flexneri*. All experiments were carried out in triplicate, and data represent the mean ± SD.

Whole plants of *A. philippense* L. (syn. *A. lunulatum* Burm. f.) were collected from the Western Ghats region of Gujarat, India (20°45′15.80″ N 73°41′42.16″ E, altitude ∼437 m) during the August–September period of 2017. The plant material was identified from its taxonomic characters as well as by a molecular sequencing method. A voucher specimen (BVBRC138) was deposited at Bapalal Vaidya Botanical Research Centre, Department of Biosciences, Veer Narmad South Gujarat University, Surat, Gujarat, India (Figures 1A–D). Collected plant material was sun dried and ground with an electric grinder into a fine powder and stored in an airtight container.

### Extraction of Plant Material

*A. philippense* powder (50 g) was soaked in methanol overnight under vigorous shaking at 110 rpm at 30°C. The methanol phase was filtered through Whatman No. 1 filter paper and concentrated using a rotary evaporator to obtain the dried residue. A total of 10% DMSO (dimethyl sulfoxide) was used to dissolve the obtained residues to make up 1000 μg/mL concentration of plant extract to perform different biological activities. *A. philippense* crude extract was soluble in 10% DMSO with little turbidity, which was properly mixed by sonication for 10 min and sterilized by filtration using a 0.25 μm filter pore size.

### Antibacterial Assays

#### Bacterial Strains

Antibacterial activity of *A. philippense* crude extract was carried out against common food pathogenic bacterial strains *E. coli* (MTCC 9537), *S. aureus* (MTCC 96), *P. aeruginosa* (MTCC 741), and *S. flexneri* (MTCC 1457). All bacterial strains were obtained from the Microbial Type Culture Collection (MTCC), Chandigarh, India, and maintained on Muller-Hinton Agar (MHA). Bacterial cultures were prepared by transferring a single colony into a fresh medium and grown overnight at 37°C. A total of 0.5 Mc Farland standard 10^8^ colony-forming units/mL (CFU/mL) were matched by adjusting the turbidity of the culture with sterile saline solution.

#### Agar Cup/Well Diffusion Method

Antibacterial activity was analyzed by an agar cup/well diffusion method on MHA. All bacterial strains were uniformly spread (100 μL) over the plates, and wells were punctured with the help of gel puncture. Into the each respective well, 100 μL of crude extract (1000 μg/mL) was inoculated, and plates were incubated at 37°C for 24 h. On the next day, the zones of inhibitions were calculated. For positive control, a chloramphenicol (1000 μg/mL) standard antibiotic was used.

### Effect of Crude Extract on Growth Curve of Bacteria

The effect of *A. philippense* crude extract on the growth curve of bacteria was observed by inoculating 0.5 mL of all grown bacterial strains individually into 150 mL of sterile nutrient broth containing 5 mL of plant extract (1000 μg/mL). A flask without a plant extract and with only a culture served as the control. Later, the growth curve was measured for each bacterial strain by taking absorbance at 660 nm at each 1 h time interval.

### Determination of Minimum Inhibitory Concentration (MIC)

MIC of *A. philippense* crude extract was performed in 96-well microtiter plates against common food pathogenic bacteria, as described previously (CLSI, 2014). The inoculums were prepared from a 6 h Muller-Hinton Broth (MHB) culture, and suspensions were adjusted to 0.5 McFarland turbidity standards (10^8^ CFU/mL). *A. philippense* crude extract was diluted to two-fold ranging from 1000 to 0.48 μg/mL (80 μL as final volume) with a DMSO concentration ≤1%. Afterward, 20 μL of bacterial suspensions and 100 μL of MHB were loaded onto microtiter plates. Plates were then incubated at 37°C for 24 h. At the end of incubation period, microtiter plates were read using spectrophotometer at 620 nm. Chloramphenicol, a standard antibiotic was used as a positive control. MHB + DMSO was used as a vehicle control, and MHB alone was used as a sterility control. MIC was recorded as the plant extract with the lowest concentration and has shown absolute inhibition of observable growth (Srinivasan et al., 2010).

### Determination of Minimum Bactericidal Concentration (MBC)

MBC was determined following the MIC assay. Wells that exhibited no evident growth had 5 μL of a sample taken and streaked on to MHA plates, and this was followed by incubation at 37°C for 18–24 h. The MBC was then recorded as the concentration at which there was minimum growth/colony of bacteria.

### Determination of Fractional Inhibitory Concentration Index (FICI)

A microdilution checkerboard test was used for determining the FICI of antibacterial combination of *A. philippense* crude extract and chloramphenicol (Lorian, 2005). For the assay, we used 96-well microtiter plates with MHB, *A. philippense* crude extract, and chloramphenicol in two-fold serial concentrations. Cell suspensions (100 μL) of respective bacterial strains, *A. philippense* crude extract (100 μL), and chloramphenicol (100 μL) were incubated at 37°C for 24 h. The FICI for the combination was assessed (Bozic et al., 2014) as:

FICI=FICofDrugA+FICofDrugB

Where

FIC A is the MIC of Drug A in the combination/MIC of Drug A alone;
FIC B is the MIC of Drug B in the combination/MIC of Drug B alone.
The amalgamation is believed to be synergistic when FICI is <0.5.
The amalgamation is believed to be additive when the FICI is >0.5 to <2.
The amalgamation is believed to be antagonistic when the FICI is > 2.

### Antibiofilm Assays

#### Assessment on Established Biofilms

The effect of *A. philippense* crude extract on biofilms was performed by the established method (Lemos et al., 2018). Biofilms of all bacterial strains were formed on 96-well microtiter plates, filled with MHB, 1% glucose, and cells (10^7^ cells/mL) for 24 h at 37°C. After the period of incubation, planktonic cells were gently discarded, and the wells were washed three times with N-saline. Then, *A. philippense* crude extract (MIC) (200 μL) was added to the wells and kept for further incubation at 37°C for 24 h. Absorbance was read at 492 nm at 0 and after 24 h. Chloramphenicol was used as a positive control. All assays were performed in triplicate. MHB medium with individual bacterial strain was used as biofilms growth control. The percentage of biofilm inhibition was estimated:

[(OD(control)-OD(test)/OD(control)]×100

#### Assessment of the Adherence of Biofilms

The effect of *A. philippense* crude extract on the inhibition of biofilm formation was accomplished by a spectrophotometric method as stated by Plyuta et al. (2013) in 96-well microtiter plates. Cell suspensions (100 μL) of respective bacterial strains (10^8^ CFU/mL) and *A. philippense* crude extract (MIC) were incubated at 37°C for 24 h. After the incubation, planktonic cells were removed by washing the wells very delicately with phosphate buffered saline (PBS) (200 μL). Biofilms developed by adherent cells were stained with 0.1% crystal violet (100 μL) followed by incubation at 37°C for 30 min. PBS was used to wash off the extra stain, and plates were then fixed with 95% ethanol (200 μL) followed by further incubation at 37°C for 15 min. Absorbance was read spectrophotometrically at 590 nm. The percentage inhibition was estimated:

[(OD(control)-OD(test)/OD(control)]×100

### Microscopic Techniques

#### Assessment of Antibiofilm Activity by Light Microscopy (LM)

Light microscopic assessment of all bacterial biofilms was accomplished by following the method of Musthafa et al. (2010) with some modifications. Overnight grown culture of all bacterial strains was added to a 5 mL freshly prepared MHB with 1% glucose. A total of 500 μL of inoculated broth (10^8^ CFU/mL) was transferred to 24-well microtiter plates containing 1 × 1 cm size cover slips. Treatment was carried out with 500 μL of the *A. philippense* crude extract (Final concentration = MIC). Chloramphenicol and sterile water of the same amount were used as positive and negative control, respectively. Biofilms on glass cover slips after incubation in a static condition for 24 h at 37°C were removed, gently washed with PBS, and stained with 0.1% crystal violet. The excess stain was washed off using de-ionized water and allowed to air dry for 5 min. Stained cover slips were observed using a LM (40x magnification) (Axioscope A1, ZEISS, Germany).

#### Assessment of Antibiofilm Activity by Fluorescence Microscopy (FM)

The biofilms of all bacterial strains were allowed to form on 1 × 1 cm size cover slips with all the respective treatments as stated above. Biofilms formed on coverslips were stained with 1% acridine orange. Excess stain was drained off, and this was followed by washing with de-ionized water and air drying for 5 min. Then, the stained cover slips were visualized under fluorescence microscopy at a magnification of 40x objectives (Axioscope A1, ZEISS, Germany).

#### Assessment of Antibiofilm Activity by Scanning Electron Microscopy (SEM)

All bacterial biofilms were analyzed by SEM (in the presence and absence of the *A. philippense* crude extract with controls against respective strains as stated above). A total of 2.5% glutaraldehyde was used for fixing the biofilms on glass coverslips for 30 min at 37°C. The fixed samples were then washed down three times with PBS and dehydrated through a graded series of 30, 50, 70, 90, and 100% of ethanol solutions for 15 min in each. Then, ethanol was reinstated with isoamyl acetate and the samples were freeze dried. Coverslips were mounted on an aluminum holder, coated with gold by an E-1010 ion sputter (Hitachi^®^), and observed under SEM (S-34002N SEM, Hitachi^®^).

### Assessment of Exopolysaccharide (EPS) Production by Ruthenium Red Staining

Ruthenium red staining assay was used for the determining the effect of *A. philippense* crude extract in diminishing EPS matrix production in all bacterial strain’s biofilm (Borucki et al., 2003). Cell suspensions (100 μL) of respective bacterial strains (10^8^ CFU/mL) and *A. philippense* crude extract (MIC) were incubated at 37°C for 24 h. After the incubation, planktonic cells were removed by washing the wells very delicately with phosphate-buffered saline (PBS) (200 μL). Biofilms developed by adherent cells were stained with 0.01% ruthenium red (Sigma-Aldrich^®^) (200 μL) to each well. Ruthenium red (200 μL) was used to fill the wells without biofilms and served as a blank, and this was followed by incubation at 37°C for 60 min. Afterward, the liquid holding the residual stain was resettled in a new microtiter plate and the absorbance was read at 450 nm. The quantity of the dye fixed to biofilms was calculated as:

$$Abs_{BF}=Abs_{B}-Abs_{S}$$

where as

Abs_B_ = absorbance of blanks
Abs_S_ = absorbance of residual stain collected from sample wells

### Identification and Analysis of Phytochemicals by High Resolution-Liquid Chromatography Mass Spectroscopy

The phytochemistry of the crude extract of *A. philippense* was analyzed using UHPLC-PDA-Detector Mass Spectrophotometer (HR-LCMS 1290 Infinity UHPLC System), Agilent Technologies^®^, United States. The liquid chromatographic system consisted of an HiP sampler, binary gradient solvent pump, column compartment, and Quadrupole Time of Flight Mass Spectrometer (MS Q-TOF) with a dual Agilent Jet Stream Electrospray (AJS ES) ion source. A total of 10 μL of the sample was injected in to the system, and this was followed by separation in SB-C18 column (2.1 × 50 mm, 1.8 μm particle size). A total of 1% formic acid in deionized water (solvent A) and acetonitrile (solvent B) were used as solvents. A flow rate of 0.350 mL/min was used, while MS detection was performed in MS Q-TOF. Compounds were identified via their mass spectra and their unique mass fragmentation patterns. Compound Discoverer 2.1, ChemSpider, and PubChem were used as the main tools for the identification of the phytochemical constituents.

### Molecular Docking Analysis of Adhesin Proteins, IcsA, Sortase A, OprD, EspA, and FimH With Phytochemicals of *A. philippense*

Crystal structures of adhesin proteins of food pathogens, IcsA from *S. flexneri* (PDB: 3ML3.pdb) (Kuhnel and Diezmann, 2011), Sortase A from *S. aureus* (PDB: 1T2P.pdb) (Zong et al., 2004), OprD from *P. aeruginosa* (PDB: 3SY7.pdb) (Eren et al., 2012), EspA from *E. coli* (PDB: 1XOU.pdb) (Yip et al., 2005), and FimH from uropathogenic *E. coli* (PDB: 1TR7.pdb) (Bouckaert et al., 2005) were fetched from Protein Data Bank (RCSBPDB). Following to the retrieval of crystal structures, LCMS identified phytochemicals three-dimensional structures, such as cholorogenic acid, caffeic acid, esculetin, rutin, coumarin, quercitrin, kaempferol, quercetin, 18-β-glycyrrhetinic acid, ursolic acid, betulin, phloroglucinol, betaine, esculin, polygodial, lagochilin, carvone, orientin, and luteolin, were acquired from eminent database PubChem and converted to PDB format using Open Babel (O’Boyle et al., 2011). These 28 compounds were then docked separately against the receptor structure (IcsA, Sortase A, OprD, EspA, and FimH) using molecular docking software Autodock 4.2.6 (Morris et al., 2009). Docking protocol was performed in a similar manner, which can be related to previous analyses (Sonawane and Barage, 2015; Parulekar and Sonawane, 2018). Apart from the grid center, all other parameters used for docking these 28 compounds with Sortase A were kept the same.

For the preparation of the grid map using a grid box, an Auto Grid (Morris et al., 2009) was used. The grid size was set to 126 × 126 × 126 xyz points for FimH and OprD receptors. For Sortase A, the grid size was set to 126 × 114 × 118 xyz points, 130 × 130 × 130 for EspA, and 104 × 126 × 96 for IcsA. Grid spacing was kept to 0.375 Å for all the receptors. The grid center for IcsA was designated at dimensions (x, y, and z) 36.394, 32.932, and 0.318; for Sortase A at (x, y, and z) -30.329, -19.713, and -0.455; for OprD at (x, y, and z): 24.439, -13.409, and 13.726; for EspA at (x, y, and z): 19.293, -4.237, and 86.963; and for FimH at (x, y, and z): 44.426, 4.358, and 31.535. The grid box is cantered in such a way that it encloses the entire binding site of both the receptors and provides enough space for translation and rotation of ligands. The generated docked conformation was ranked by predicted binding energy and topmost binding energy docked conformation was analyzed using UCSF Chimera (Pettersen et al., 2004) for intermolecular hydrogen bonding of active site amino acid residues from the receptors with docked ligands (Nadaf et al., 2018).

### Statistical Analysis

Results are represented as mean values with standard error. Statistical analyses were executed using ANOVA test followed by Bonferroni to compare the controls and treated groups at a significance level of 5% with GraphPad Prism Version 7.03 software.

## Results

### Qualitative Phytochemical Screening

The percent yield of the extract was 6.89%, i.e., 68.89 mg/g dry wt. of whole plant (w/w). Preliminary phytochemical scrutiny of methanolic crude extract of *A. philippense* revealed the existence of phenolics, terpenoids, alkaloids, tannins, flavonoids, glycosides, saponins, and carbohydrates (Table 1).

**TABLE 1:** Phytochemicals of *A. philippense* crude extract.

Phytochemicals	Crude extract
Alkaloids	+
Flavonoids	+
Terpenoids	+
Steroids	-
Glycosides	+
Phenolics	+
Saponins	+

*Key: + = present, – = absent.*

### Antibacterial Potential of *A. philippense*

**FIGURE 2:**
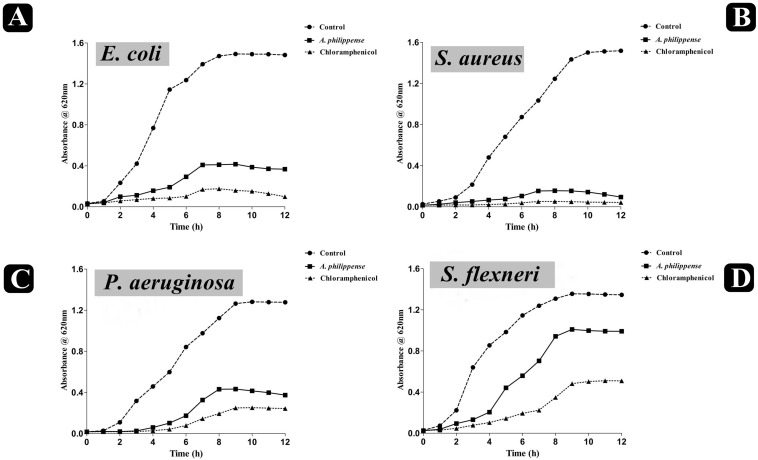
Growth curve analysis of bacteria. **(A)** Growth curve pattern of *E. coli*, with and without plant extract; **(B)** growth curve pattern of *S. aureus*, with and without plant extract; **(C)** growth curve pattern of *P. aeruginosa*, with and without plant extract; and **(D)** growth curve pattern of *S. flexneri*, with and without plant extract. Chloramphenicol was used as positive control.

The antagonistic potential of crude extract of *A. philippense* was studied using agar cup/well diffusion method against food pathogens (*S. aureus, P. aeruginosa*, *E. coli*, and *S. flexneri*). Antibacterial activity results are portrayed in the form of zone of inhibition and revealed substantial antagonistic activity against all the four tested bacterial strains. *S*. *aureus* was found to be more susceptible when compared to *E. coli*, *P. aeruginosa*, and *S*. *flexneri* (Figure 1E). Results of growth curve analysis displayed the efficacious inhibition of all tested bacterial strains. In contrast to control, the growth of all bacterial strains demonstrated a delayed lag phase and protracted logarithmic phase (Figures 2A–D).

### Determination of MIC, MBC, and FICI

**FIGURE 3:**
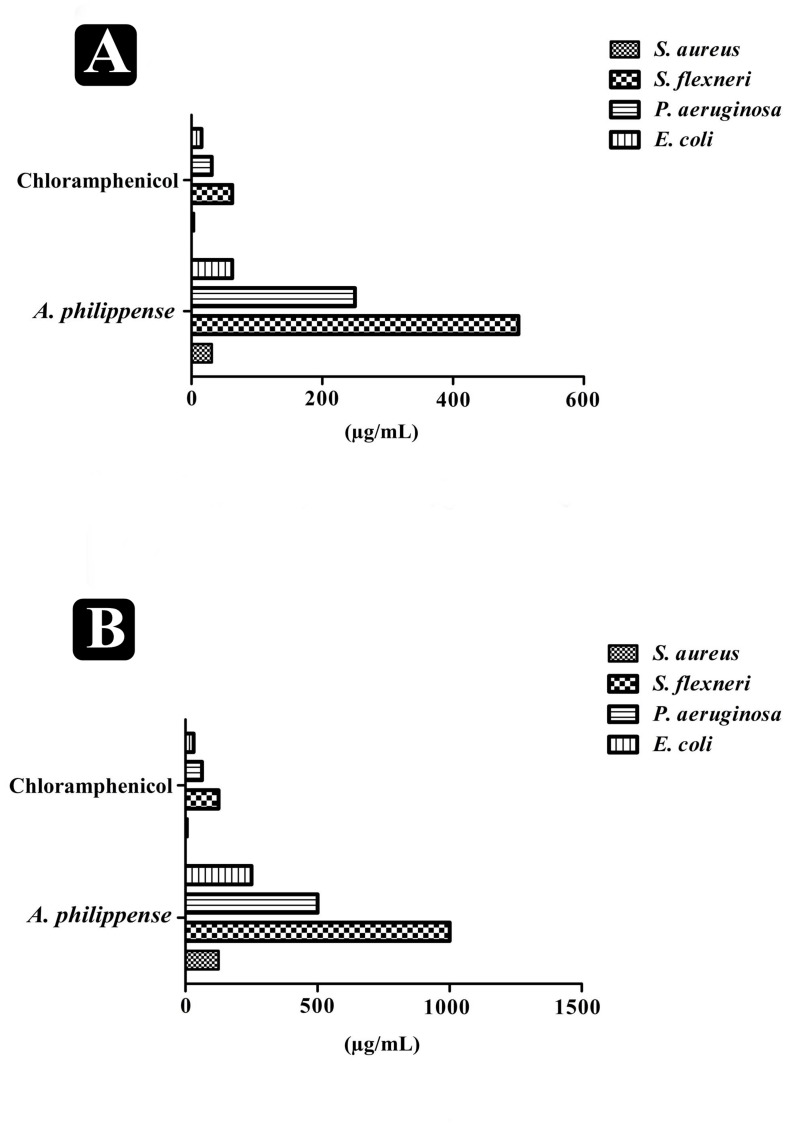
Antagonistic potential of *A. philippense* crude extract. **(A)** Results of MIC of *A. philippense* crude extract and chloramphenicol; and **(B)** results of MBC of *A. philippense* crude extract and chloramphenicol.

The antibacterial potential of *A. philippense* crude extract was assessed by determining the MIC and MBC against tested foodborne pathogens. Chloramphenicol, a broad-spectrum antibiotic, was used as a positive control because it is commonly used to treat infections caused by the tested pathogens. *A. philippense* crude extract displayed a broad-spectrum antagonistic potential and effectual against tested Gram-negative and Gram-positive bacteria. MIC displays the minimum concentration of antimicrobial agent which remarkably inhibits growth, whereas MBC displays the minimum concentration of antimicrobial agent prompting the microbial death. An assessment of MBC can be an excellent and comparatively economical tool to concurrently assess many antimicrobial agents for effectiveness. Antibacterial compounds are generally considered as bactericidal on condition that MBC is no more than four times the MIC (Lemos et al., 2018). Corresponding to MIC values, *E. coli, S. aureus*, *P. aeruginosa*, and *S. flexneri* were the most susceptible to *A. philippense* crude extract (Figures 3A,B). The values of MIC and MBC was about 62.5 and 250 μg/mL for *E. coli*, 31.25 and 125 μg/mL for *S. aureus*, 500 and 1000 μg/mL for *S. flexneri*, and 250 and 500 μg/mL for *P. aeruginosa*. The values of MIC and MBC are also represented in Table 2. These results are evidence enough to prove the bactericidal potential of phytochemicals present in *A. philippense* crude extract. For both *A. philippense* and chloramphenicol, the checkerboard assay showed a decline in the MIC values. This clearly suggests a plausible interaction between each other and exhibited a significant result of synergistic action between both *A. philippense* and chloramphenicol for all tested organisms except *S. flexneri* (Table 3).

**TABLE 2:** Antibacterial activity of *A. philippense* crude extract.

*A. philippense* crude extract (μg/mL)			Chloramphenicol (μg/mL)	
Bacterial strain	MIC	MBC	MIC	MBC
*E. coli*	62.5	250	15.65	31.25
*S. aureus*	31.25	125	3.9	7.812
*S. flexneri*	500	1000	62.5	125
*P. aeruginosa*	250	500	31.25	62.5

**TABLE 3:** FICI determination of *A. philippense* crude extract.

Bacterial strain	*A. philippense*	Chloramphenicol	FICI	Effect
	MIC*	MIC*		
*E. coli*	8.928	4.471	0.428	Synergy
*S. aureus*	4.464	1	0.399	Synergy
*S. flexneri*	83.333	31.25	0.666	Additive
*P. aeruginosa*	41.666	9.765	0.479	Synergy

* MIC in a combination of A. philippense and Chloramphenicol (μg/mL). FICI evaluated as: synergistic when, FICI is <0.5; additive when the FICI is >0.5 to <2 and antagonistic when the FICI is >2.

### Effect on Adhesion and Established Biofilms

**FIGURE 4:**
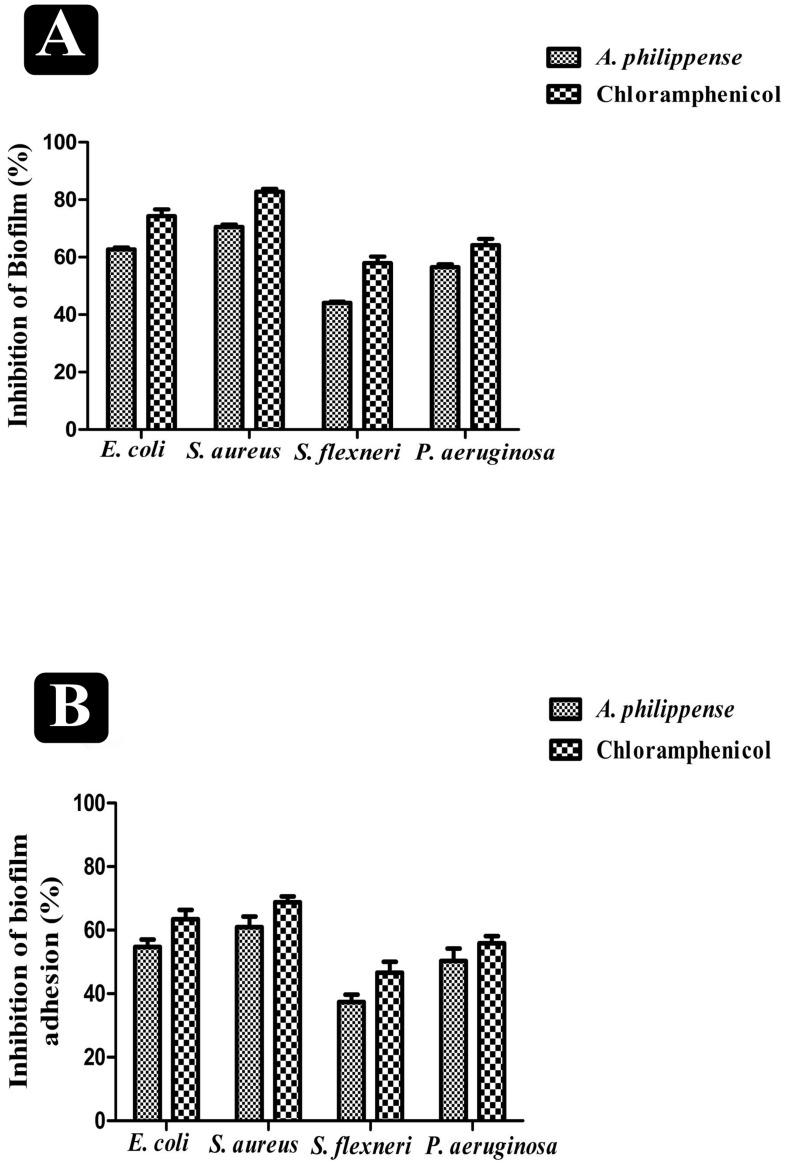
Antibiofilm potential of *A. philippense*. **(A)** Effect of *A. philippense* crude extract on established biofilms of *E. coli*, *S. aureus*, *P. aeruginosa*, and *S. flexneri*; and **(B)** effect of *A. philippense* crude extract on adherence ability of *E. coli*, *S. aureus*, *P. aeruginosa*, and *S. flexneri*. Chloramphenicol was used as positive control. All experiments were carried out in triplicate, and data represent the mean ± SD.

*A. philippense* crude extract was capable enough to distort the preformed biofilms and have an impact on their adhesion ability and was assessing at the MIC level. Obtained results revealed that *A. philippense* had an affinity to hinder the growing and preformed biofilms by hampering their adhesion potentiality at MIC. At this concentration, the inhibition of preformed biofilms by *A. philippense* was about 62.72% for *E. coli*, 70.58% for *S. aureus*, 44.10% for *S. flexneri*, and 56.54% for *P. aeruginosa*. *A. philippense* was also found to decrease the adhesion ability of biofilms with percentage of inhibition as 54.73% for *E. coli*, 60.92 for *S. aureus*, 37.34% for *S. flexneri*, and 50.26 for *P. aeruginosa* (Figures 4A,B).

### Visualization of Disrupted Biofilms by Microscopic Analysis (LM, FM, and SEM)

**FIGURE 5:**
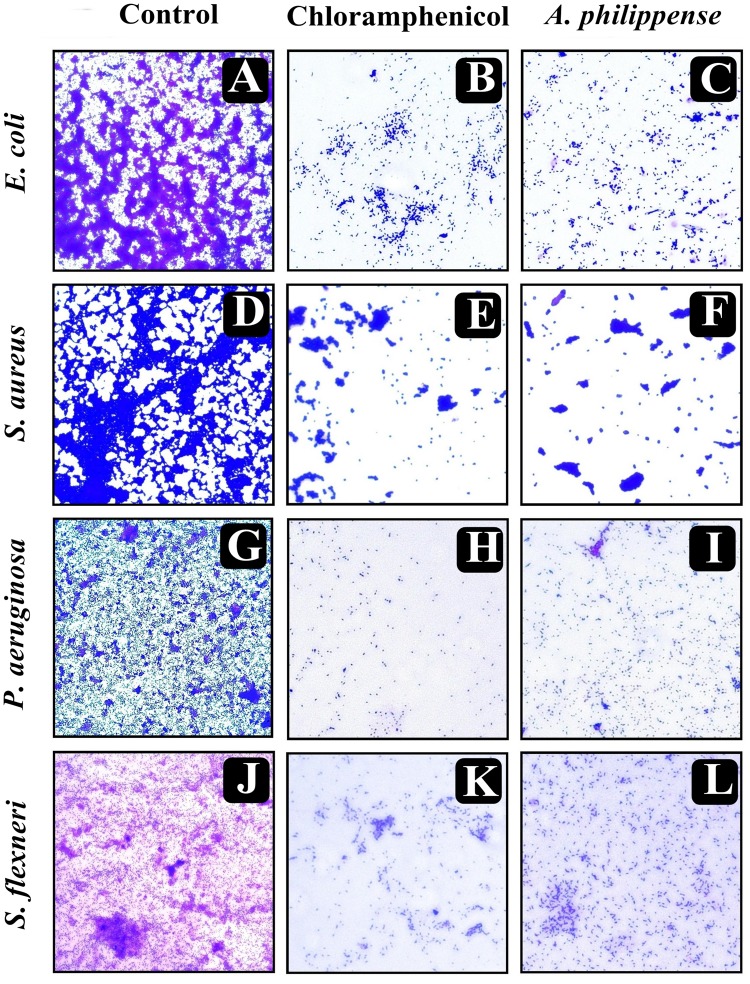
Micrographs of disrupted matured biofilms of tested strains formed on glass surfaces by the *A. philippense* crude extract at their respective MICs by light microscopy (at 40x magnification). **(A,D,G,J)** Growth control; **(B,E,H,K)** Positive control chloramphenicol; and **(C,F,I,L)** *A. philippense* crude extract.

**FIGURE 6:**
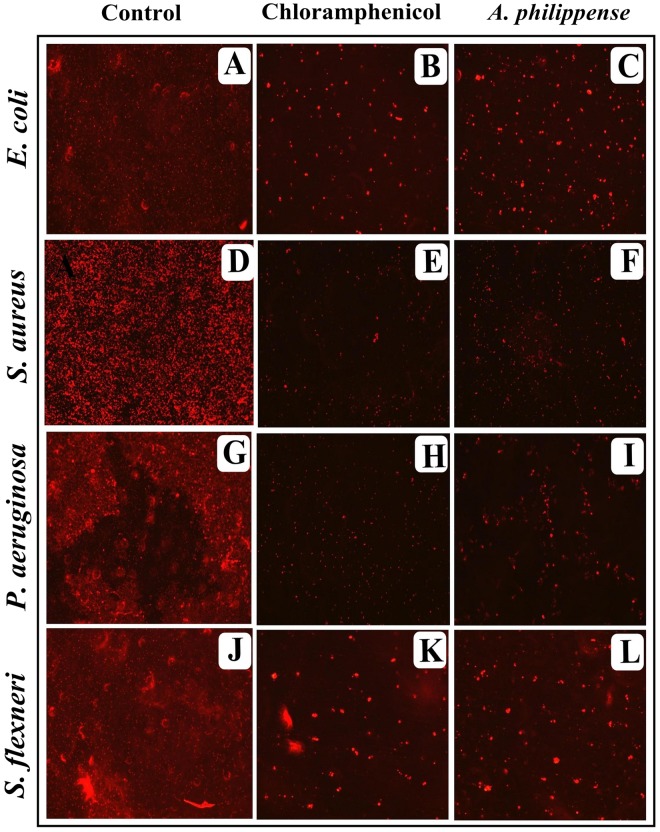
Micrographs of disrupted matured biofilms of tested strains formed on glass surfaces by the *A. philippense* crude extract at their respective MICs by fluorescent microscopy (at 40x magnification). **(A,D,G,J)** Growth control; **(B,E,H,K)** Positive control chloramphenicol; and **(C,F,I,L)** *A. philippense* crude extract.

**FIGURE 7:**
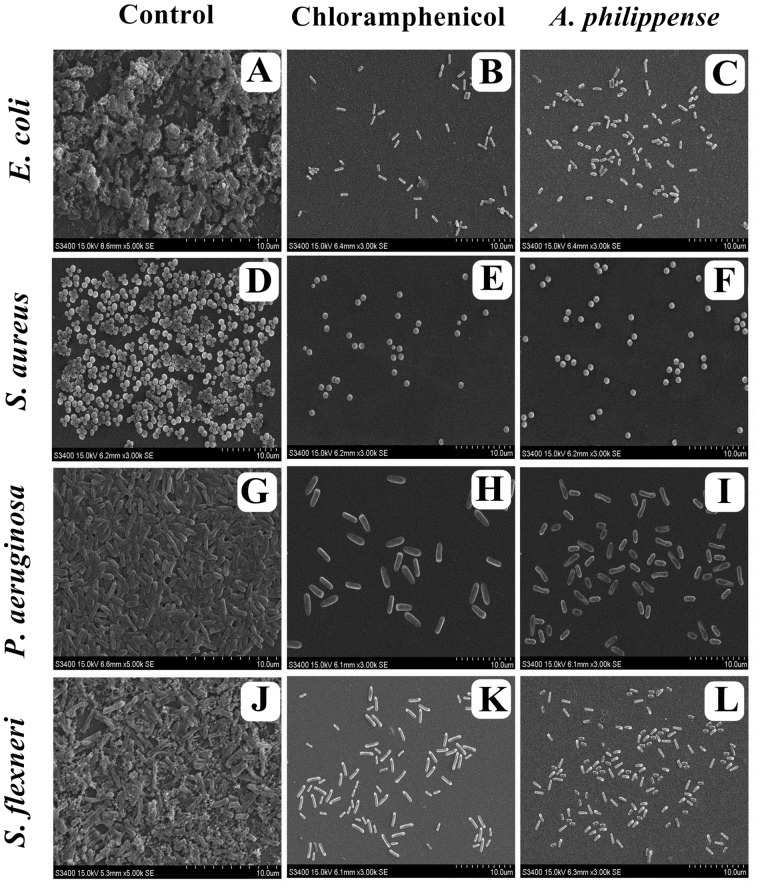
Micrographs of disrupted matured biofilms of tested strains formed on glass surfaces by the *A. philippense* crude extract at their respective MICs by scanning electron microscopy. **(A,D,G,J)** Growth control; **(B,E,H,K)** Positive control chloramphenicol; and **(C,F,I,L)** *A. philippense* crude extract.

In the first instance, light and fluorescence microscopy were used as direct microscopic methods to gather the evident information on treated biofilms. In LM, crystal violet was applied to stain the matured biofilms formed on glass cover slips to analyze the effect of *A. philippense* at its MIC. Heavy-knit like mat of biofilms was appeared under microscope in control, whereas biofilms were appeared to reduce with nominal appearance of micro colonies in the presence of *A. philippense* extract (Figures 5A–L). The antibiofilm potential of *A. philippens*e crude extract described above was additionally confirmed by acridine orange staining in FM. Results of FM also showed a scattered emergence of extract treated samples compared with the control (Figures 6A–L). In the second instance, SEM analysis was also done to study the surface morphology and anatomy of biofilms formed by different food pathogenic bacteria with or without *A. philippense* crude extract. Prototypical multi-tiered growth of biofilms was observed in the control group, while the chloramphenicol-treated group displayed a notable lessening in the amount of biofilms. Exceptionally, *A. philippense* also led to a noteworthy reduction in biofilm formation by tested food pathogens (Figures 7A–L). Our results have provided evidence to support that *A. philippense* has an effective antimicrobial potential against the different foodborne pathogens and can, at the same time, inhibit cell adhesion, which will ultimately control the formation of biofilms.

### EPS Production

**FIGURE 8:**
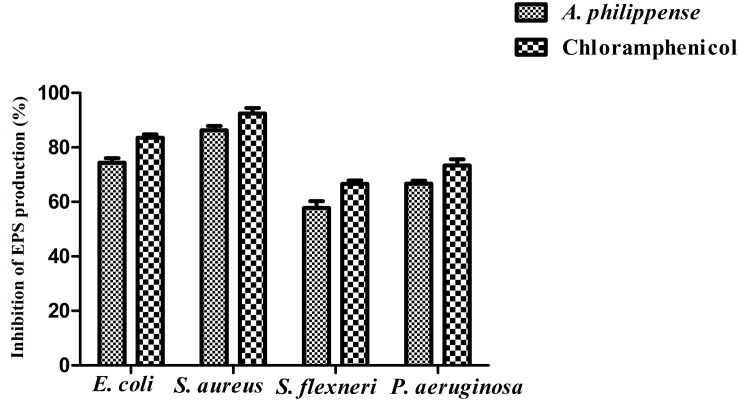
Result of total EPS production inhibition (%) by different bacterial strains in the presence of *A. philippense* crude extract at their respective MICs. Chloramphenicol was used as positive control. All experiments were carried out in triplicate, and data represent the mean ± SD.

Bacterial cells inside the biofilms produce EPS, aiding the entrapment of nutrients and also functioning as a mean of defense (Jayathilake et al., 2017). In the present study, total EPS production was remarkably decreased in all food pathogens treated with *A. philippense* crude extract at MIC. In contrast to control, EPS production in *E. coli* and *S. aureus* lowered by 74.40 and 86.31%, respectively, whereas, in *S. flexneri* and *P. aeruginosa*, it decreased by 57.75 and 66.73%, respectively (Figure 8).

### Bioactive Compounds Present in *A. philippense* Crude Extract

**FIGURE 9:**
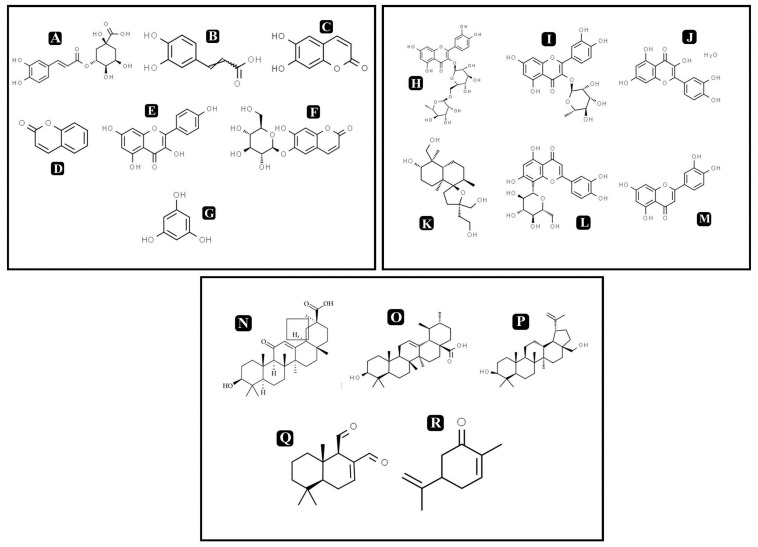
Chemical structures of identified compounds by HR-LCMS **(A)** chlorogenic acid, **(B)** caffieic acid, **(C)** esculetin, **(D)** coumarin, **(E)** kaempferol, **(F)** chlorogenic acid, phloroglucinol, **(G)** esculin, **(H)** rutin, **(I)** quercitrin, **(J)** quercetin, **(K)** lagochilin, **(L)** orientin, **(M)** luteolin, **(N)** 18-β-glycyrrhetinic acid, **(O)** ursolic acid, **(P)** betulin, **(Q)** carvone, and **(R)** polygodial.

On the basis of significant antibacterial and antibiofilm potentials, crude extract of *A. philippense* was used for studying the phytochemistry of its constituents by using HR-LCMS. With the detailed Mass spectra data, absorbance spectra, and retention times compared with the available literature, the chemical composition of *A. philippense* holds different bioactive compounds (Table 4). Phenolic compounds, such as chlorogenic acid, caffeic acid, esculetin, coumarin, kaempferol, phloroglucinol, and esculin; flavonoid compounds, such as rutin, quercitrin, quercetin, lagochilin, orientin, and luteolin; and terpenoid compounds, such as 18-β-glycyrrhetinic acid, ursolic acid, betulin, polygodial, and carvone, were, to the best of our knowledge, reported for the first time from *A. philippense* (Figure 9).

**TABLE 4:** Identified major phytochemicals by HR-LCMS showing antibiofilm activity from *A. philippense* crude extract.

Compounds	Formula	Class of phytochemicals	*m/z*	RT (min)	Mass	Mode of action	References
Chlorogenic acid	C_16_H_18_O_9_	Phenol	357.15	2.569	354.09444	Biofilm Inhibition *Salmonella* typhimurium	Tang et al., 2015
Caffeic acid	C_9_H_8_O_4_	Phenol	188.06	2.271	180.04202	Biofilm Inhibition *Salmonella* typhimurium	Tang et al., 2015
Esculetin	C_9_H_6_O_4_	Phenol	172.10	4.665	178.02634	–	–
Rutin	C_27_H_30_O_16_	Flavonoids	612.14	8.194	610.15246	Biofilm Inhibition *Salmonella* typhimurium	Tang et al., 2015
Coumarin	C_9_H_6_O_2_	Phenol	142.42	8.521	146.03661	QS and Biofilm inhibition *E. coli* O157:H7	Girennavar et al., 2008; Reen et al., 2018
Quercitrin	C_21_H_20_O_11_	Flavonoid	466.72	9.016	448.09999	–	–
Kaempferol	C_15_H_10_O_6_	Phenol	284.19	9.585	286.04735	Biofilm inhibition *Salmonella*, *Shigella dysenteriae*, *Shigella flexneri*, *Shigella sonnei* and *Escherichia coli*	Kurkin and Sharova, 2007; Kabra et al., 2019
Quercetin	C_15_H_10_O_7_	Flavonoid	301.05	10.351	302.04222	Inhibition of gene involved in biofilm production -Biofilm Inhibition *Salmonella* typhimurium	Lee et al., 2014; Tang et al., 2015
18-β-Glycyrrhetinic acid	C_30_H_46_O_4_	Terpenoid	478.36	19.344	470.33901	Biofilm inhibition *Streptococcus mutans*	Yu et al., 2018
Ursolic acid	C_30_H_48_O_3_	Terpenoids	448.16	22.338	456.35961	Biofilm Inhibition *Salmonella* typhimurium	Tang et al., 2015
Betulin	C_30_H_50_O_2_	Triterpene	441.37	22.4	442.38024	Biofilm inhibition *Streptococcus pyogenes*	Viszwapriya et al., 2016
Phloroglucinol	C_6_H_6_O_3_	Phenol	124.57	6.439	126.03167	–	
Esculin	C_15_H_16_O_9_	Phenol	338.16	2.974	340.07909	–	
Polygodial	C_15_H_22_O_2_	Terpenoids	236.16	16.436	234.16165	Biofilm inhibition *Candida* sp.	(Santos et al., 2017)
Lagochilin	C_20_H_36_O_5_	Flavonoids	364.04	12.8	356.25583	–	
Carvone	C_10_H_14_O	Terpenoids	169.45	8.959	150.10434	Biofilm inhibition *Staphylococcus aureus*	Nostro et al., 2007; Porfirio et al., 2017
Orientin	C_21_H_20_O_11_	Flavonoid	458.46	7.787	448.09999	–	
Luteolin	C_15_H_10_O_6_	Flavonoids	280.07	10.31	286.04735	Biofilm Inhibition *Salmonella* typhimurium	Tang et al., 2015

### Molecular Docking

**FIGURE 10:**
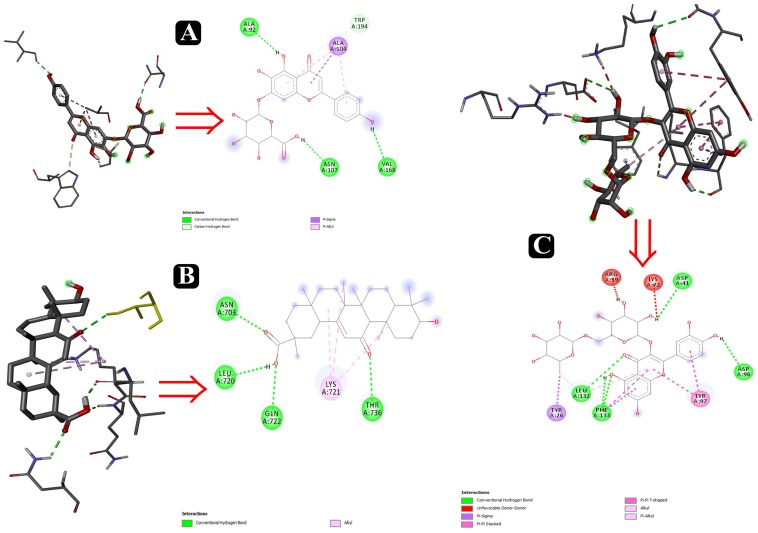
Interactions of adhesin proteins with phytochemicals. **(A)** 1T2P from *S. aureus* with higher binding affinity antibiofilm agent scutellarin. Interaction of active site amino acid residue VAL 168, ASN107, TRP194, ALA104, and ALA92 from 1T2P with scutellarin; **(B)** 3ML3 from *S. flexneri* with higher binding affinity antibiofilm agent 18-β-glycyrrhetinic acid. Interaction of active site amino acid residue ASN703, LEU720, GLN722, and THR736 from 3ML3 with 18-β-glycyrrhetinic acid; and **(C)** 3SY7 from *P. aeruginosa* with higher binding affinity antibiofilm agent rutin. Interaction of active site amino acid residue TYR26, LEU132, PHE133, ASP41, ASP96, LYS72, and ARG39 from 3SY7 with rutin.

**FIGURE 11:**
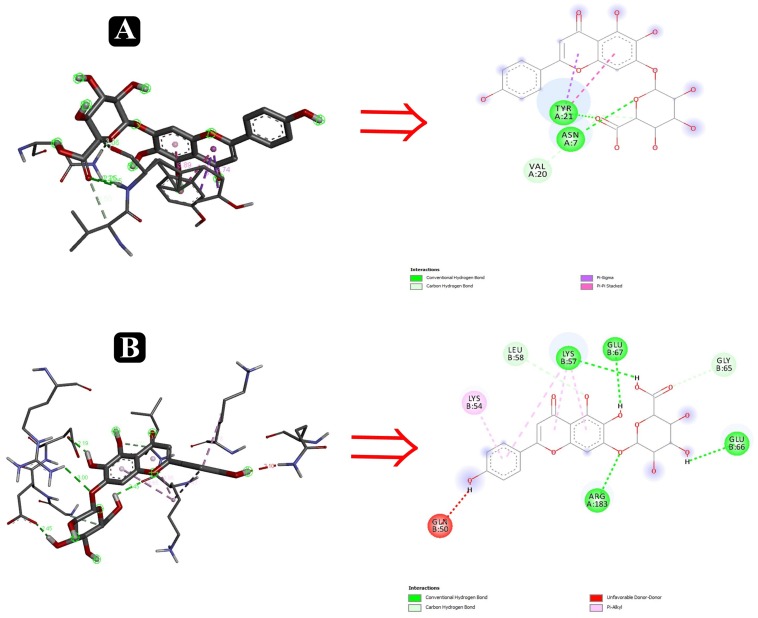
Interactions of adhesin proteins with phytochemicals. **(A)** 1TR7 from *E. coli* with higher binding affinity antibiofilm agent scutellarin. Interaction of active site amino acid residue ASN7, VAL20, and TYR21 from 1TR7 with scutellarin; and **(B)** 1XOU from *E. coli* with higher binding affinity antibiofilm agent scutellarin. Interaction of active site amino acid residue ARG183, GLN50, GLN66, GLU67, LEU58, and LYS57 from 1XOU with scutellarin.

To predict the mode of binding and affinity between receptor and linker, AutoDock Vina^®^ was used to couple the phytochemicals identified via HR-LCMS from *A. philippense* crude extract and adhesion proteins. The high affinity of the compounds for the protein was represented by lower binding energy. Molecular coupling of the adhesion proteins associated with the compounds showed that all compounds have a high binding affinity. Binding affinities of the top-rated pose of ligand–receptor complex is represented in Table 5. Compounds occupied the active site in different ways and can be observed in Figures 10, 11.

**TABLE 5:** Binding affinities of top-rated pose of ligand–receptor complex.

Compound name	1T2P	3ML3	3SY7	1XOU	1TR7
Chlorogenic acid	−6.4	−5.8	−7.8	−6.7	−6.9
Betulin	−6.3	−5.6	−8.7	−5.5	−5.8
Coumarin	−5.8	−4.9	−6	−5.5	−5.8
Ursolic acid	−6.4	−6.8	−9.3	−6.2	−7.3
Rutin	−6.7	−7.2	−9.5	−7.8	−7.8
Kaempferol	−6.7	−6.5	−7.6	−6.7	−6.9
18−β−Glycyrrhetinic acid	−6.3	−7.3	−8.7	−6.1	−7.6
Scutellarin	−7.3	−7.2	−9	−8	−8.2
Caffeic acid	−5.1	−4.7	−5.9	−4.9	−5.7
Quercitrin	−7.1	−6.7	−8.7	−6.2	−7.4
Esculetin	−5.3	−5.4	−6.5	−5.7	−5.5
Phloroglucinol	−4.6	−4	−4.9	−4.2	−5.3
Quercetin	−6.8	−6.6	−7.3	−6.9	−6.9
Betaine	−3.4	−3.2	−3.7	−3.2	−3.8
Esculin	−6.4	−6	−7.3	−5	−5.9
Polygodial	−5.5	−5.2	−6	−5.5	−6
Lagochilin	−5.3	−5.3	−6.7	−6.4	−5.9
Carvone	−4.6	−4.5	−5.4	−4.2	−5.5
Orientin	−7.2	−6.6	−8.6	−7	−7
Luteolin	−6.7	−6.5	−7.8	−5.8	−7

*Binding affinity measured in kcal/mol.*

## Discussion

Nowadays, foodborne bacteria have become an increasing matter of concern around the world, as they are the highest cause of severe foodborne diseases. In turn, this poses a great risk for food industries and health safety. Diverse bacterial species are capable of growing on various types of food surfaces, including those related to food industry infrastructures, no matter what material the infrastructure is made of Zhao et al. (2017). Due to different environmental conditions in distinct food industries, bacteria are forced to transform into using the biofilm form of life. Biofilm-producing bacteria are resistant to antibiotics and any other chemical or environmental fluctuation inside the biofilms, as opposed to their planktonic form (Song et al., 2019). This resistance to antimicrobial agents by bacteria inside biofilms is a crucial issue for food industries, as issues surrounding this tender huge economic losses to this sector. Loss by biofilms is not only confined to food industries – its impact is undeniably worse to marine and oceanic industries through damaging to ship hulls (Blando et al., 2019). Thus, there is an urgent need to consider biofilms as a target for pharmacological development, and new strategies are required to control the biofilm mode of growth.

Currently, natural products are the center of attention for researchers and may prove the effectiveness of secondary metabolites in fighting against the biofilms (Adnan et al., 2018). Natural products are valued to be the safest, as they are derived from natural sources (Adnan et al., 2017a,c) and do not affect the surfaces and surroundings of biofilms while acting upon them. Therefore, in the search of a natural antibacterial and antibiofilm compounds that are profoundly required to act on food pathogenic bacteria, we have selected *A. philippense*. The crude extract of this medicinal fern showed broad-spectrum antibacterial activity (Reddy et al., 2001) and was found to be enormously effective against both planktonic and biofilms forms of food pathogenic bacteria. We have exemplified a potent inhibitory activity of this plant against *E. coli*, *S. aureus*, *P. aeruginosa*, and *S. flexneri*. Hence, this study provides proof of the ethanomedicinal usage of *A. philippense* in the treatment of variety of diseases and infections caused by pathogenic microorganisms.

In the context of antibacterial remedies, drug amalgamation has loads of advantages in comparison to the use of single agents. This may be in the form of achieving synergistic activity, to impede the emergence of resistant bacteria, and to lower the side effects because of the use of lower drug concentration (Ocampo et al., 2014). The amalgamation of *A. philippense* crude extract and chloramphenicol was imperative to optimize the antibacterial efficacy of both. Moreover, futuristic studies are necessitating to test antibacterial resistance toward other drugs.

The bacterial growth curve can examine the growth and death of bacteria above a broad range of antibacterial concentrations and has been frequently used to evaluate the effect of antibacterial agents over time (Khan et al., 2014; Foerster et al., 2016). When the concentration of antibacterial agents exceed MIC for the bacteria, a time-dependent bactericidal effect occurs (Anantharaman et al., 2010). Our growth curve analysis demonstrated the time-dependent bactericidal effects of *A. philippense* crude extract for all tested food pathogens. Comparing the effect of *A. philippense* crude extract with chloramphenicol standard antibiotic, we noted similar action begin at starting hours (Figures 2A–D). Time-dependent killing of all selected bacterial strains by *A. philippense* crude extract indicated that the antibacterial activity could be the result of a variety of physiological factors within the cell (Saritha et al., 2015). Moreover, the growth of all tested food pathogenic bacteria in the presence of *A. philippense* crude extract was indicated by a delayed lag phase and a slow logarithmic phase, when compared to control. Therefore, additional studies are needed to investigate the potential of the plant extracts to exert influence on the cellular events, such as the repression of macromolecular synthesis.

The antibiofilm efficiency of *A. philippense* crude extract was displayed against both Gram-negative and Gram-positive foodborne bacterial strains. Biofilm production was recorded by all tested pathogens. *A. philippense* crude extract shown exceptional efficiency in inhibiting the biofilms of all tested strains at their respective MICs in a concentration dependent manner. However, one remarkable finding resulting from the current study was the proficiency of *A. philippense* crude extract in distorting the preformed biofilms as well as obstructing the adhesion for different bacterial strains (Figures 4A,B). A standard crystal violet and acridine orange assay intended for evaluating the biofilm biomass showed that *A. philippense* crude extract was more efficient in the extermination of preformed biofilms formed by all tested pathogens. This was further confirmed by SEM analysis by decreasing the multilayer growth of biofilms and free living cells by influencing the integrity of cell wall. Additionally, it was also observed that the disturbed cell wall of all bacterial strains led to failure in the emergence of cluster and incapable of maintaining their typical morphology in presence of the extract. The development of biofilm begins with the initial adhesion step with the aid of EPS, which upon maturation, forms a typical shape (Hall-Stoodley et al., 2004). On the other hand, quorum sensing (QS) is the another chief action involving in the biofilm formation, where, microbial cells can communicate to each other through signaling molecules and has been extensively studied in bacteria for controlling the biofilms (Rutherford and Bassler, 2012). It has been described that the inhibitory activity of plant secondary metabolites on biofilm development and on QS is an event that relies on density and requires proper crowdedness of the microbial cells (Nazzaro et al., 2013). Nevertheless, our results delineate that the biofilm development can be prevented at early stage by inhibiting the adhesion, which can help in designing novel therapeutic strategies.

Few previously reported studies have revealed that phytochemicals were involved in prevention of biofilms by inhibiting adhesion via different mechanisms. Plant extracts have been proven to have the exceptional capability of impeding the first stage of biofilm development by six bacterial strains via interfering with the attachment forces like Lifshitz–Van der Waals, Brownian, sedimentation, and electrostatic interaction forces, which promotes bacterial attachment to various types of surfaces (Roy et al., 2018). Not only do the plant extracts interfere with the attachment, they may also hinder the accessibility of organic, inorganic, and other nutrients that are necessary for the adhesion and bacterial cell growth (Sandasi et al., 2010). Another study reported that ethanolic and acetone crude extract of *Psidium guajava* blocks the adhesion of *Streptococcus mutans* (Razak and Rahim, 2003). Similar results were seen in a study that reported the potent anti-adhesion action of *E. brasiliensis, E. leitonii*, *E. involucrate*, and *E. myrcianthes* leaf extracts against *C. albicans* (Sardi et al., 2017).

The EPS matrix is a significant make-up of biofilms that forms gel-like structures, is exceedingly hydrated, and has a three-dimensional charged environment in which the microbial cells are basically restrained (Adnan et al., 2010, 2017c). Results of the present study revealed that *A. philippense* crude extract carried out the inhibition of EPS in all tested bacterial strains. Reduction in the biochemical constitution of the biofilm matrix weakens the complexity of biofilm and make it easy for the drugs to access (Lentino, 2003). Altogether, our data demonstrated that *A. philippense* restricts the formation of biofilms.

Bioactive compounds known to have antibiofilm potential with other medicinal importance from the *A. philippense* crude extract were identified via HR-LCMS analysis (Table 4). From the identified phytochemicals, phenolic compounds like chlorogenic acid and caffeic acid are well-known vital antioxidants with a few important properties, such as anti-obesity, anti-inflammatory, anti-neoplastic, anticancer, and antibiofilm activity against *S. typhimurium* (Cho et al., 2010; Rajendra Prasad et al., 2011; Tang et al., 2015); coumarin has been reported as a potent antibiofilm agent that inhibits biofilm formation in *E. coli* O157:H7 via interfering with QS system (Girennavar et al., 2008). Polygodials have also been proven to exhibit antimicrobial, anti-hyperalgesic, anti-inflammatory, and anti-allergic activities (da Cunha et al., 2001; Martin et al., 2009). Kaempferol has been reported to exhibit a wide range of pharmacological activities – antimicrobial, antioxidant, anticancer, anti-inflammatory, anti-diabetic, anti-osteoporotic, anxiolytic, analgesic, anti-allergic activities, and antibiofilm activity – against varieties of bacterial species of *Salmonella* sp., *S. dysenteriae, S. flexneri S. sonnei*, and *E. coli* (Kim et al., 2005; Kurkin and Sharova, 2007; Calderon-Montano et al., 2011; Kabra et al., 2019).

Betulin and polygodial are terpenoids with antimicrobial, antitumor, anti-hyperalgesic, anti-inflammatory, antiallergic, anti-obesity, and antibiofilm properties against *S. pyogenes* and *Candida* sp., respectively (da Cunha et al., 2001; Alakurtti et al., 2006; Martin et al., 2009; Tang et al., 2011; Viszwapriya et al., 2016). 18-β-glycyrrhetinic acid is a terpenoid with anti-inflammatory, anti-allergic, antiulcer, antioxidant, anti-microbial, and antibiofilm properties against *S. mutans* (Yu et al., 2018). Ursolic acid has antibiofilm potential against *S. typhimurium*, and carvone has been reported as an insect repellent and an antibacterial, antifungal, antioxidant, and antibiofilm agent (Tang et al., 2015). From the detected flavonoids, rutin has been reported for its antibiofilm activity against varieties of bacterial species of *Salmonella* sp., *S. flexneri, E. coli*, and *S. aureus* (Kabra et al., 2019).

Quercetin has also been reported as a potent antibiofilm agent with a potential against biofilms of different bacterial species, such as *E. coli*, *S. aureus*, *S. typhimurium*, etc. (Murakami et al., 2008). Luteolin has a potent apoptosis-inducing and chemo-preventive activities. It induces apoptosis and direct cell cycle arrest in tumor cells. It also inhibits cell proliferation and suppresses metastasis (Kandaswami et al., 2005; Adnan et al., 2017b). Different medicinal plants have been reported earlier to inhibit biofilms of different bacteria that contains these types of phytochemicals especially via targeting quorum sensing (Yang et al., 2009).

After the identification of phytochemicals, results were elaborated upon by attempting a molecular docking analysis, and it was detailed to the atomic level. It is well known that biofilm formation by foodborne pathogens is a multi-step process in which adhesion plays the most important and influential role. Five well-studied proteins – Sortase A, IcsA, OprD, EspA, and FimH from *S. aureus*, *S. flexneri*, *P. aeruginosa*, and *E. coli* – help in the first step of the biofilm formation process by attaching to the host tissues. Deterring the movement of these proteins will therefore eventually inhibit the process of biofilm formation and, ultimately, their virulence factors.

Several compounds, such as scutellarin, 18-β-glycyrrhetinic acid, and rutin, have been identified from the molecular docking (Table 5). These recognized compounds are associated with the parent antibiofilm compounds (Catechin, Eugenol, Apigenin, Emodine, Umbelliferone, Esculetin, and Quercetin) and could play an imperative role in antibiofilm activity (Magesh et al., 2013; Lee et al., 2014; Muruzovic et al., 2016). Consequently, our molecular docking results also elucidated the function of LCMS-identified compounds as inhibitors of *S. aureus*, *S. flexneri*, *P. aeruginosa*, and *E. coli* biofilms. Initially, SEM analysis also revealed the same (Figures 10, 11).

Altogether 28 compounds were identified and they all showed the appropriate binding mode at the active site of Sortase A (Ala92, Ala104, Asn107, Val168, and Trp194), IcsA (Asn703, Leu720, Gln722, and Thr736), OprD (Tyr26, Arg39, Asp41, Lys72, Asp96, Leu132, and Phe133), EspA (Gln50, Lys57, Leu58, Glu66, Glu67, and Arg183), and FimH (Asn7, Val20, and Tyr21) (Table 6). Proper intermolecular hydrogen bonding interactions was seen during the substrate binding of all the identified antibiofilm compounds with the active site of Sortase A, IcsA, OprD, EspA, and FimH proteins (Zong et al., 2004; Lin et al., 2014). The residue Tyr121 from FimH of *E. coli*, Phe133 and Arg39 from OprD of *P. aeruginosa*, Gln50, Glu66, and Glu67 from EspA of *E. coli*, Leu720 from IcsA of *S. flexneri*, and Ala92 and Asn107 from Sortase A of *S. aureus* formed strong interactions with the antibiofilm agents with standard hydrogen binding pattern (Tables 5, 6). As a result, interactions of these key residues with antibiofilm agents could possibly help in inhibiting the crucial step of biofilm forming process, i.e., adhesion.

**TABLE 6:** Interacting active site residues of receptors with different antibiofilm agents.

Receptor–ligand	Receptor ligand interactions	Distance in angstroms
1T2P–Scutellarin	(VAL168) CO—HO (Ligand)	2.45
	(ASN107) OD1—HO (Ligand)	2.13
	(TRP194) CD1—OC (Ligand)	3.58
	(ALA104) CB—benzopyran (Ligand) – Pi-sigma interaction	3.87
	(ALA92) CO—HO (Ligand)	2.28
3ML3–18-Beta Glycyrrhetinic acid	(ASN703) HD21—OC (Ligand)	2.36
	(LEU720) CO—HO (Ligand)	2.22
	(GLN722) NH—OC (Ligand)	2.42
	(THR736) HG1—OC (Ligand)	2.51
3SY7–Rutin	(TYR26) phenyl ring—CC (Ligand) – Pi-sigma interaction	3.90
	(LEU132) NH—OC (Ligand)	2.93
	(PHE133) CO—HO (Ligand)	2.20
	(PHE133) NH—O (Ligand)	2.62
	(ASP41) OD1—HO (Ligand)	2.88
	(ASP96) CO—HO (Ligand)	2.64
	(LYS72) ZN3—HO (Ligand)	2.35
	(ARG39) NH11—HO (Ligand)	1.16
1XOU–Scutellarin	(ARG183) H–O (Ligand)	3.00
	(GLN50) NH–HO (Ligand)	2.10
	(GLU66) CO–OH (Ligand)	2.45
	(GLU67) CO–OH (Ligand)	2.19
	(LEU58) C–O (Ligand)	3.80
	(LYS57) OH–OC (Ligand)	2.45
1TR7–Scutellarin	(ASN7) NH–O (Ligand)	2.06
	(VAL20) C–OC (Ligand)	3.50
	(TYR21) NH–OC (Ligand)	2.21
	(TYR21) CO–CO (Ligand)	3.19
	(TYR21) pi-pi interaction	3.80
	(TYR21) pi-pi interaction	3.89
	(TYR21) Phenyl ring–CO interaction	3.74

Collectively, the present study revealed for the first time that *A. philippense* contains a diverse group of phytochemicals that exhibit extensive antibacterial potential against all assessed Gram-positive and Gram-negative foodborne bacteria. This potency could be due to the targeting of a variety of physiological factors within the cell, such as the production of macromolecules and membrane destabilization. *A. philippense* was also able to inhibit biofilms of bacteria via hampering production of EPS. Amalgamation of *A. philippense* with chloramphenicol could be utilized to diminish the bacterial resistance and ameliorate the treatments against the infections caused by these foodborne pathogens except *S. flexneri*. Moreover, the results of *in silico* docking analysis would be useful to propose new lead compounds against biofilm producing pathogenic foodborne bacteria.

## Data Availability Statement

The datasets generated for this study are available on request to the corresponding author.

## Author Contributions

MAd, MP, MR, and VD performed the conceptualization and design. MP, AS, MAl, NE, and VD performed the data curation. MAd, MP, SD, AS, MR, and MAl performed the formal analysis. VD, MP, MR, NE, and AS, carried out the methodology. SD, MP, and MAd used the software. MAd, MP, SD, AS, and MAl carried out the validation. MP, AS, MAl, and VD carried out the investigation. MP, SD, and MAd wrote the original draft. MAd, MP, SD, AS, MR, MAl, NE, and VD wrote, reviewed, and edited the manuscript.

## Conflict of Interest

The authors declare that the research was conducted in the absence of any commercial or financial relationships that could be construed as a potential conflict of interest.
